# Biliary Cyst: An Unusual Cause of Cholestasis Post Cholecystectomy

**DOI:** 10.7759/cureus.53171

**Published:** 2024-01-29

**Authors:** Mark Ayoub, Carol Faris, Tiana Dodd, Shawn Chillag

**Affiliations:** 1 Internal Medicine, West Virginia University School of Medicine, Charleston, USA; 2 Internal Medicine, Charleston Area Medical Center, Charleston, USA; 3 General Surgery, Marshall University, Huntington, USA

**Keywords:** post cholecystectomy complications, jaundice cholestatic, biliary cysts, right upper quadrant abdominal pain, hepato biliary cancers, roux-en-y hepaticojejunostomy, obstructive jaundice, magnatic resonance cholangiopancreaticography(mrcp), endoscopy ercp, choledochal cysts

## Abstract

Biliary cysts are relatively uncommon and they can be congenital or acquired and can have various presentations such as cholelithiasis, cholangitis, jaundice, and pancreatitis. Biliary cysts are associated with a high risk of biliary cancers and such risk increases with age. Identification of biliary cysts warrants an aggressive approach to lower cancer risk. Surgical management has a high success rate and it lowers morbidity, mortality, and cancer risk. We present a 40-year-old female who had a cholecystectomy in 2016. She presented with obstructive jaundice and was found to have a class I biliary cyst. She underwent endoscopic retrograde cholangiopancreatography with stenting which led to complete resolution of her symptoms. Later, she underwent elective Roux-en-Y hepaticojejunostomy with cyst resection three months later. She underwent a successful recovery.

## Introduction

Biliary cysts are defined as dilated parts within the biliary tract and are about 1% of benign biliary diseases [1]. Biliary cysts are more common in females but still relatively uncommon, with the incidence in Western populations being 1:100,000 [2,3]. Cysts may be congenital or acquired. Cysts are more commonly present in children before age 10, however, up to 20% of the cases are found in adults [2]. Patients with biliary cysts have a higher risk of biliary cancer as they advance in age. Some studies suggest that biliary tract malignancy occurs in more than 10% of patients in their third decade of life [4-6]. Cholangiocarcinoma is the most fatal complication of biliary cysts; however, various other complications were reported such as cholelithiasis, cholangitis, jaundice, and pancreatitis [7-10]. Identification of biliary cysts requires prompt surgical intervention to prevent such complications, which usually has a high success rate.

## Case presentation

A 40-year-old female without significant past medical history but with a surgical history of cholecystectomy in 2016 presented to the emergency room with a one-month history of progressively worsening nausea and abdominal pain. She reported that the pain was located in the right upper quadrant (RUQ), intermittent in nature, and occurred after meals. She did not have any alcohol use history and did not take any medications on a regular basis.

On physical exam, she was tachycardic with a heart rate in the 100s and normotensive. She appeared comfortable; however, jaundice and scleral icterus were noted. No palmar erythema or spider angiomas were seen. She had RUQ tenderness to deep palpation; however, Murphy’s sign was negative. Otherwise, her abdomen was soft with positive bowel sounds with no rebound or guarding.

Initial lab work evaluation showed some abnormalities as highlighted in Table 1. Her alkaline phosphatase (ALP) level was elevated at 330 U/L, however, ALT and AST were within normal range. Her total bilirubin was elevated at 7.6 mg/dl with a direct component of 4 mg/dl which was also elevated. Her lipase level was normal at 49 U/L. The remainder of her lab results were unremarkable.

**Table 1:** Table showing lab values on the day of admission and the day of discharge ALP: alkaline phosphatase; ALT: alanine transaminase; AST: aspartate aminotransferase

Labs (normal range)	On admission	On discharge
Serum lipase (0-160 u/L)	49 u/L	-
ALP (40-129 u/L)	330 u/L	136 u/L
ALT (7-55 u/L)	24 u/L	15 u/L
AST (8-48 u/L)	40 u/L	21 u/L
Total Bilirubin (0.3-1 mg/dL)	7.6 mg/dL	1.7 mg/dL
Direct bilirubin (0.1-0.3 mg/dL)	4 mg/dl	-

**Figure 1:**
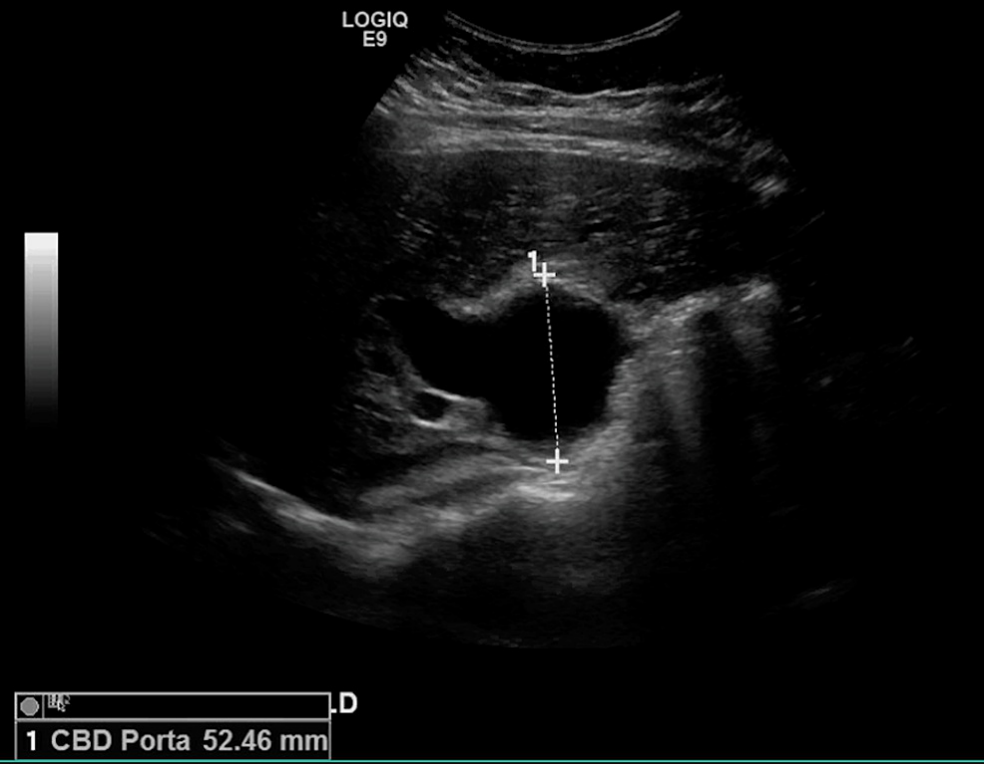
RUQ US showing significantly dilated CBD RUQ: right upper quadrant; US: ultrasound; CBD: Common bile duct

A RUQ ultrasound (US) was performed which showed significant dilatation of the common bile duct (CBD) at 5.2 cm and findings concerning for a class IV choledochocele and intrahepatic duct dilation as seen in Figure 1.

**Figure 2:**
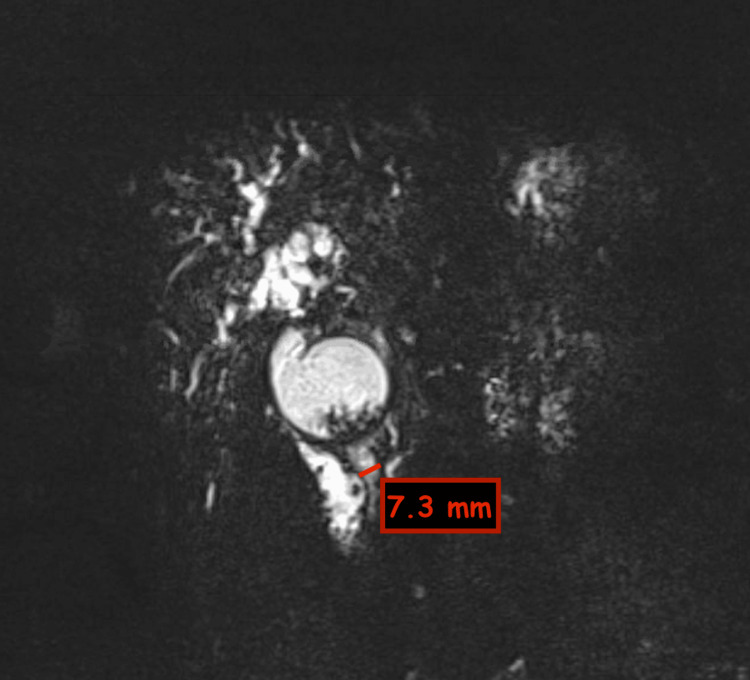
Coronal MRCP section showing significantly dilated CBD MRCP: magnetic resonance cholangiopancreatography; CBD: common bile duct

**Figure 3:**
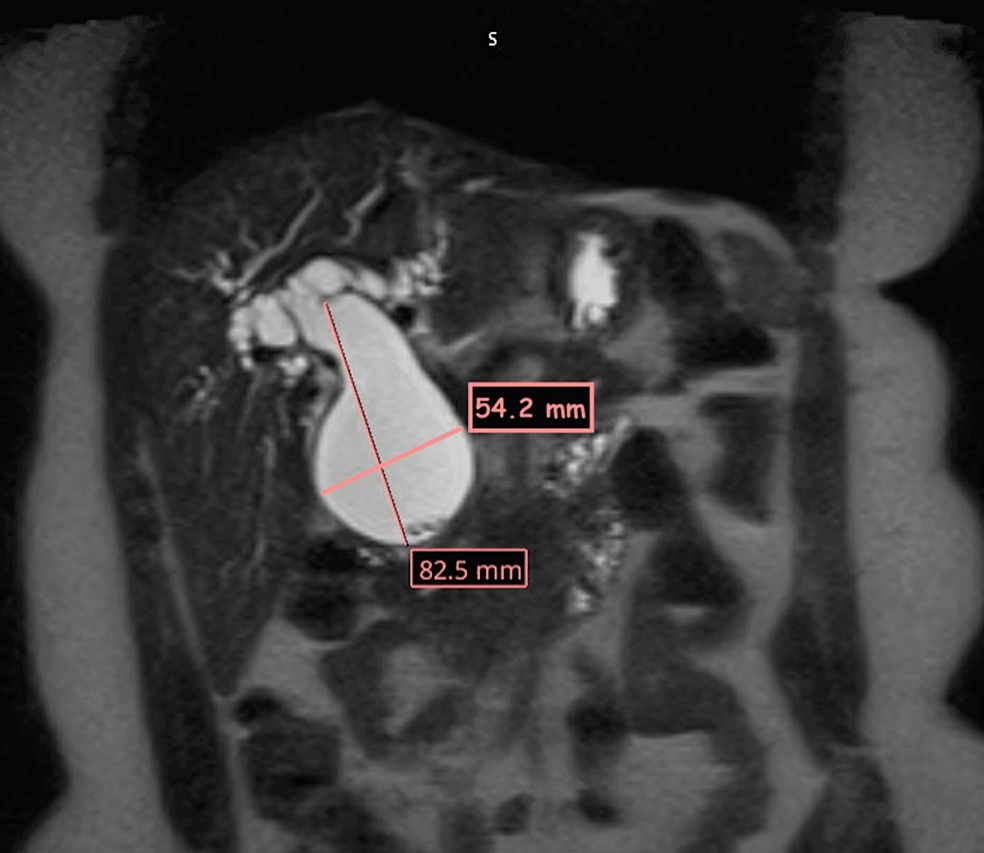
Coronal MRCP section showing biliary cyst with stone-like material MRCP: magnetic resonance cholangiopancreatography

**Figure 4:**
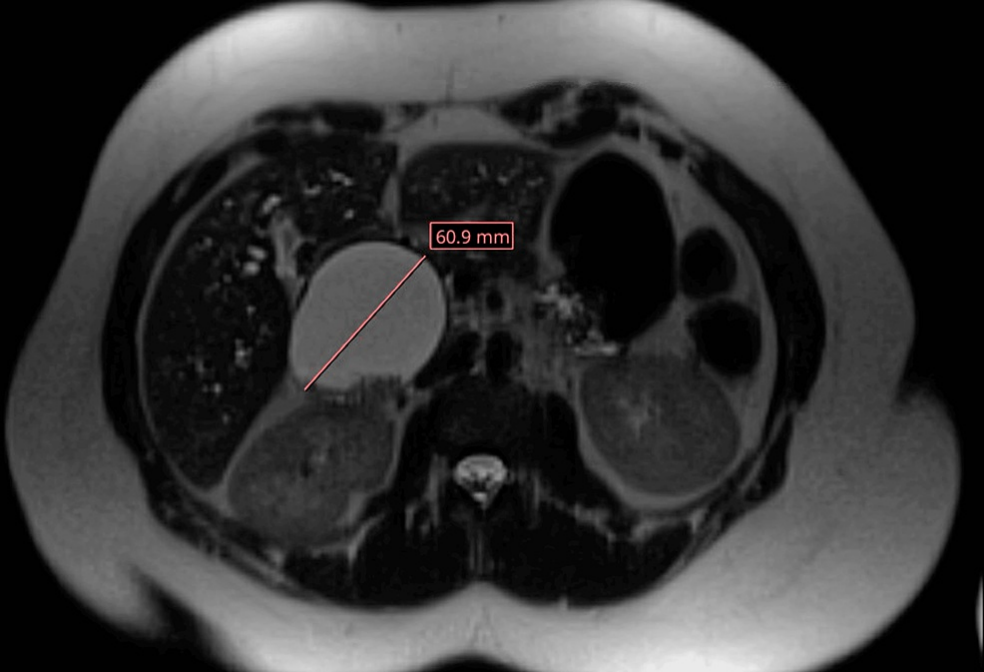
Axial MRCP section showing biliary cyst with prominent intrahepatic bile ducts MRCP: magnetic resonance cholangiopancreatography

**Figure 5:**
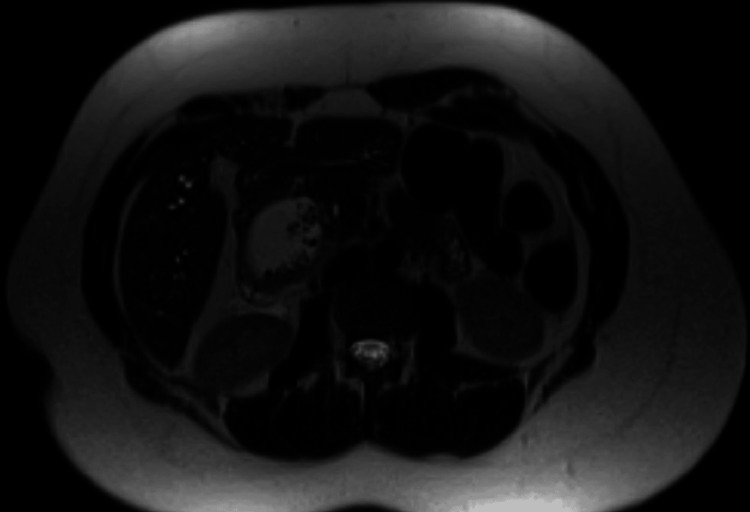
Axial MRCP section showing stone-like material within the biliary cyst MRCP: magnetic resonance cholangiopancreatography

**Figure 6:**
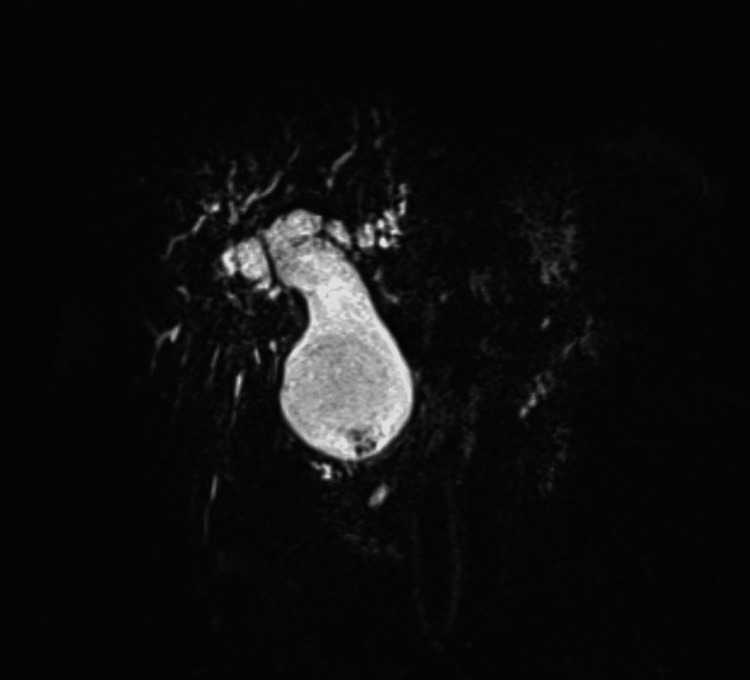
MRCP showing the biliary tree with a biliary cyst, intrahepatic ducts, and stone-like material within the cyst MRCP: magnetic resonance cholangiopancreatography

A magnetic resonance cholangiopancreatography (MRCP) was performed and revealed progressive CBD dilatation with worsening intrahepatic biliary dilation. The choledochal cyst appeared fusiform in shape and was markedly distended, measuring 6 cm x 8.2 cm and containing stone material. MRCP findings are highlighted in Figures 2-6.

She was admitted with obstructive jaundice and underwent urgent endoscopic retrograde cholangiopancreatography (ERCP) with biliary sphincterotomy, balloon sweeps of the bile ducts, and placement of a plastic pigtail pancreatic stent prophylactically as well as a therapeutic biliary stent. ERCP also revealed that her cyst was class I and not class IV as seen on imaging as in Figure 8. 

**Figure 7:**
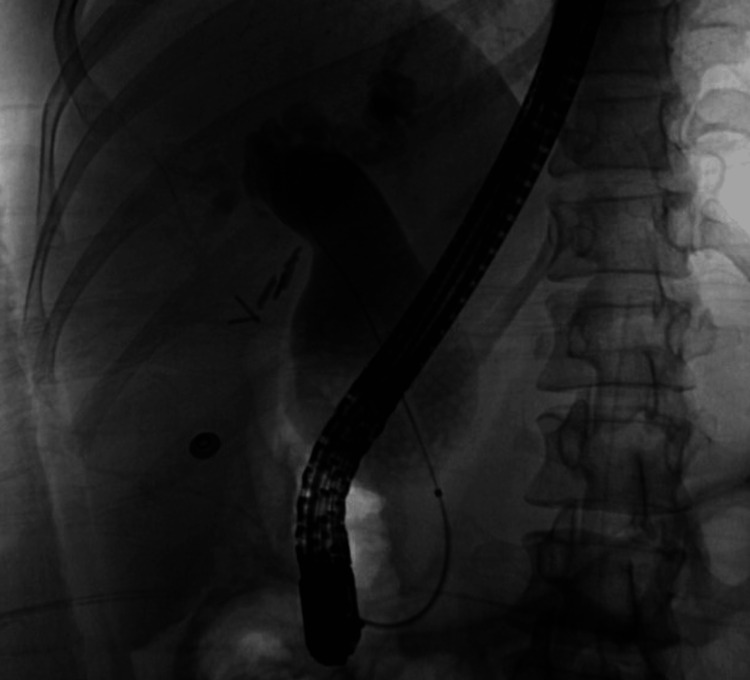
ERCP showing dilated biliary cyst with some faint filling defects resembling stone-like material within the cyst ERCP: endoscopic retrograde cholangiopancreatography

CBD brushing and fluid analysis did not show any malignant cells. Her jaundice and pain resolved after the endoscopic intervention and she was eventually discharged home. She later underwent Roux-en-Y hepaticojejunostomy and choledochal cyst resection three months after her discharge and had a successful recovery. Intraoperative assessment showed that the CBD was grossly dilated and easily located. It also showed numerous stones within the CBD. Recently, her surgical resection pathology came back showing a choledochal cyst wall with inflamed granulation tissue and reactive columnar epithelial nests consistent with a choledochal cyst. There was no evidence of malignancy. 

Of note, her initial cholecystectomy was done in 2016 due to symptomatic cholelithiasis. RUQ US at that time showed a gallbladder that was completely filled with sludge and stones with no wall thickening or CBD dilation. The surgical report did not show any dilation at that time and postoperative pathology revealed cholelithiasis with findings of chronic cholecystitis. 

## Discussion

Biliary cysts are dilatation of the biliary tract that can cause various symptoms. Typically, biliary cysts are benign and they comprise 1% of benign biliary diseases [1]. The incidence of biliary cysts in Western countries is reported to be 1:100,000 to 1:150,000 [2,3]. Biliary cysts can be congenital or acquired and they are associated with biliary cancer. The risk of biliary cancer is reported to be more than 10% in patients with biliary cysts in their third decade of life [4-6]. The risk of cancer increases with advanced age [8]. Biliary cysts can also have other complications such as cholelithiasis, cholangitis, jaundice, and pancreatitis [5,7-10].

**Figure 8:**
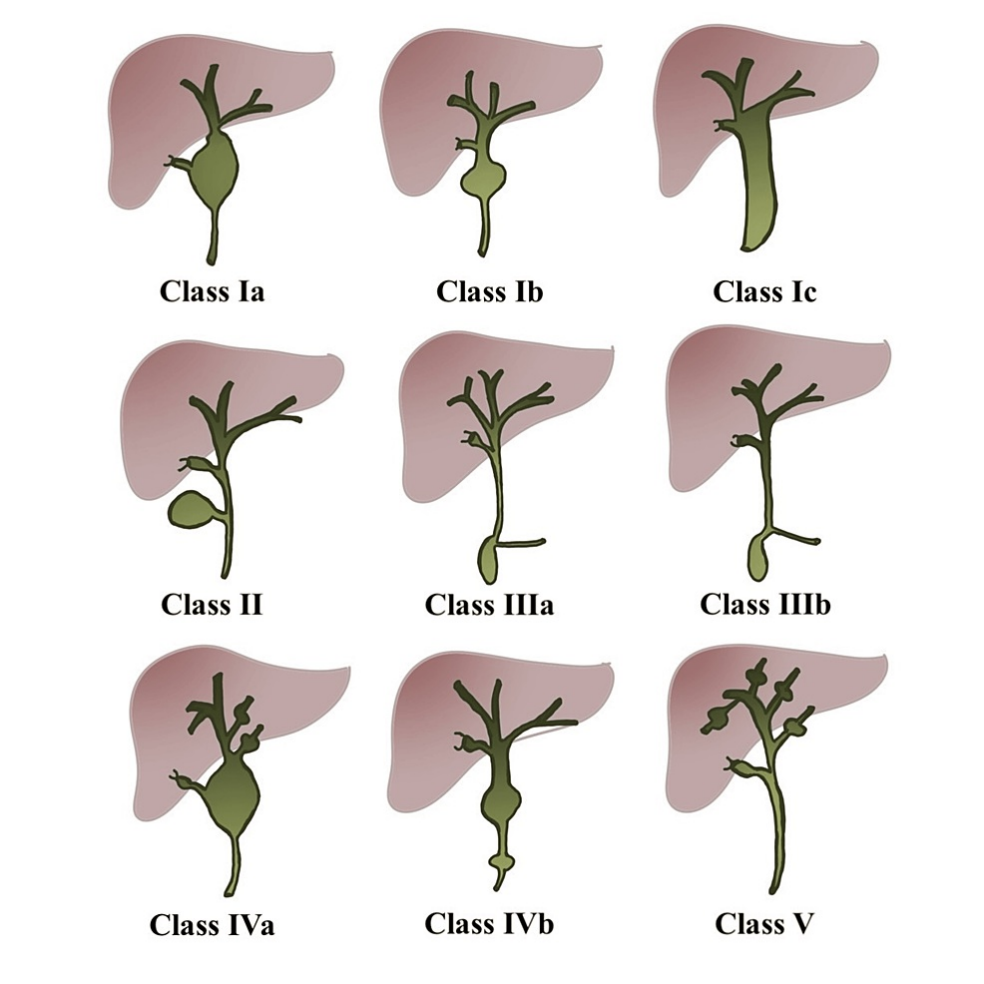
Illustration of Todani classification of biliary cysts This image was created by the authors.

Todani classified biliary cysts into five major categories as in Figure 8 [11,12]. Todani classification was initially used in congenital biliary cysts, however, adult biliary cysts have been increasingly reported. Radiology literature expanded the modified Todani classification to allow it to be used in adults and is commonly used in their imaging reports [13]. Class I with solitary extrahepatic cyst which occurs in 40-85% of the cases. This can be further divided into IA: common type; IB: segmental extrahepatic duct dilation; and IC: diffuse extrahepatic dilatation. Class II with extrahepatic diverticulum which occurs in 2-3% of the cases. Class III with choledochocele which occurs in 1.4-5.6% of the cases. Class IV occurs in 18-20% of the cases and is further divided into IVA with multiple extra- and intra-hepatic cysts and IVB with multiple extrahepatic cysts. Class V with multiple intrahepatic cysts and this class is rare. 

Multiple histopathological types of cancers associated with biliary cysts were reported. Adenocarcinoma was most common with up to 84%. The most common locations of cancer were the extrahepatic bile duct up to 62%, gallbladder up to 46%, and intrahepatic bile duct up to 2.5% [8,11,14]. Todani et al. were able to report the cancer incidence with each class. Around 68% of cancers were associated with class I, 5% with class I, 1.6% with class III, 21% with class IV, and 6% with class V [15].

With such a high risk of biliary cancer in patients with biliary cysts, prompt management is warranted. Total excision is recommended to avoid occurrence. Roux-en-Y hepaticojejunostomy has a success rate of 92% with a complication rate of 7% [16]. According to the literature, the incidence of malignancy drops postoperatively to 0-6% [17,18]. Patients who undergo surgical resection require postoperative surveillance and monitoring for both postoperative complications and cancer occurrence risk.

Our patient underwent cholecystectomy for symptomatic cholelithiasis. At that time, there was no evidence of biliary dilation. It is believed that the reason for her presentation at this time is multifactorial; postoperative changes after her initial cholecystectomy along with the presence of multiple biliary stones. This combination led to significant dilation of her biliary tree and her subsequent presentation with obstructive jaundice. This was further confirmed by the presence of numerous stones during both ERCP and Roux-en-Y procedures and was also seen in the postoperative pathology report.

Our patient was initially thought to have a class IV biliary cyst. Further workup revealed that it was class I. Class I is associated with the highest cancer risk, therefore, surgical resection with Roux-en-Y hepaticojejunostomy was recommended and offered to her to decrease such risk. She eventually had a successful recovery.

## Conclusions

Biliary cysts can be congenital or acquired and are highly associated with biliary cancer. They can have various clinical presentations such as cholelithiasis, cholangitis, jaundice, and pancreatitis. Cancer risk varies depending on the etiology of the cyst and that risk increases with advancing age. Cancer risk was also noted to differ between different classifications therefore prompt classification of the biliary cysts helps dictate patients’ care. Early surgical resection is proven to lower cancer risk, morbidity, and mortality and is often recommended. Surgical resection is usually tolerated and is often successful. 
